# Role of unilateral‐cannulating adrenal venous sampling for the subtyping of primary aldosteronism for adrenalectomy: Experience from a low‐volume center

**DOI:** 10.1002/wjs.12402

**Published:** 2024-11-17

**Authors:** Chi‐Man Tom Chow, Man Sze Carol Lai, Xina Lo, Yuk Wah Shirley Liu

**Affiliations:** ^1^ Department of Surgery Alice Ho Miu Ling Nethersole Hospital Hospital Authority Hong Kong China; ^2^ Division of Endocrine Surgery Department of Surgery the Chinese University of Hong Kong Hong Kong China

**Keywords:** adrenal glands, catheterization, hyperaldosteronism

## Abstract

**Background:**

Current guidelines recommend adrenal venous sampling (AVS) for subtyping primary aldosteronism (PA). However, bilateral adrenal cannulation failure is common especially at low‐volume centers. The role of unilateral‐cannulating AVS in selecting patients for unilateral adrenalectomy is unclear.

**Methods:**

Fifty consecutive patients underwent AVS between 2009 and 2023 and thirty‐three (66%) underwent unilateral adrenalectomy. We defined unilateral PA (uPA) by the normalized plasma aldosterone and the aldosterone–renin ratio following unilateral adrenalectomy. We examined the effectiveness of unilateral‐cannulating AVS in identifying uPA.

**Results:**

88%, 50%, and 48% of patients had the left, right, and bilateral adrenal veins successfully cannulated, respectively. Among patients with bilateral successful cannulation, a lateralization index (LI) > 4 provided a sensitivity of 100% and a positive predictive value (PPV) of 86.7% for uPA. Thresholds for the contralateral suppression index (CSI) and relative aldosterone secretion index (RASI) were derived from this subgroup and applied to the entire cohort. CSI <0.5 demonstrated a sensitivity of 76.5% and PPV of 92.9% for uPA, whereas RASI >2.4 showed a sensitivity of 85.0% and PPV of 94.4% for uPA. With CSI <0.5 or RASI >2.4 combined, a higher PPV (95.5%) was achieved when compared to computed tomography and iodocholesterol scintigraphy (86.2% and 62.5%, respectively).

**Conclusion:**

Despite unsuccessful bilateral cannulation, our study confirms that unilateral‐cannulating AVS can effectively select patients for unilateral adrenalectomy based on a combination of CSI or RASI criteria.

## 1 INTRODUCTION

Unilateral primary aldosteronism (uPA) has a high rate of cure following adrenalectomy. Hence, correctly identifying uPA is essential. ^1^ Methods distinguishing unilateral from bilateral PA included posture stimulation testing, iodocholesterol scintigraphy, aldosterone‐potassium ratio, clinical prediction models, and adrenal volumetric assessments. ^2^, ^3^, ^4^, ^5^, ^6^ However, these methods were neither accurate nor reproducible. Adrenal computed tomography (CT) is crucial for assessing the presence of nodules and excluding carcinoma. However, CT is limited for subtyping because nonfunctioning incidentalomas are common, micronodules may be undetected, and bilateral nodularities are often encountered. A systematic review analyzing the use of CT for subtyping found that 15% of patients received nonindicated surgery, 4% were operated on the wrong side, and 20% missed out on the opportunity of surgical cure. ^7^

Adrenal venous sampling (AVS) is an accurate subtyping modality for uPA with sensitivity and specificity of 95% and 98.6%, respectively, by expert centers. ^8^ Although guidelines recommend AVS as the gold standard investigation for subtyping uPA, its widespread adoption was limited. In a large‐scale multinational cohort, only 13% of patients received AVS for the workup for PA. ^9^ This underutilization was attributed to the belief that AVS is technically challenging, invasive, and rarely successful. ^10^ The success rate of AVS was correlated to expertise. Expert centers have reported bilateral successful cannulation rates of up to 96%. ^11^ However, a German registry study demonstrated that the success rate was only 8%–10% in low‐volume centers compared to a national standard of 31%. ^12^ A learning curve study reported the need for 36 cases of AVS to reach proficiency and 27 cases per year to maintain expertise. ^13^

Conventionally, unilateral‐cannulating AVS results were considered uninformative, leading to clinical dilemmas on repeating AVS, resorting to other subtyping methods, or proceeding with surgery. Theoretically, unilateral PA (uPA) could be confirmed when the contralateral normal adrenal suppression is identified or when excessive aldosterone is demonstrated to be derived predominantly from one side. ^11^, ^14^ This study aims to evaluate the accuracy of utilizing unilateral‐cannulating AVS results to select patients for adrenalectomy.

## 2 MATERIAL AND METHODS

***Study design, setting, and data collection:*** A retrospective study was conducted between January 2009 and December 2023 involving all consecutive patients with AVS for the subtyping of PA at our university center in Hong Kong. Demographical, clinical, biochemical, and radiological data were reviewed in the electronic medical records for the diagnosis of PA until 1 year following AVS or adrenalectomy.

This study was approved by the local institutional review board and followed the Declaration of Helsinki. Reporting was made according to the Strengthening the Reporting of Observational Studies in Epidemiology (STROBE) guidelines.

***Inclusion/exclusion criteria:*** This study included all patients with PA who underwent AVS. PA was diagnosed according to the Endocrine Society clinical practice guidelines. ^1^ The plasma aldosterone‐to‐renin ratio (ARR) was used to screen for potential cases of PA. Patients with elevated ARR >750 pmol/L per ng/mL/h underwent further confirmation by oral salt loading or saline infusion tests where a 24‐h urinary aldosterone level of >33 nmol/d was diagnostic for PA. All patients diagnosed with PA underwent CT to evaluate adrenal nodules and exclude carcinomas. Exclusion criteria of the present study were patients who declined AVS, patients with contrast allergy, patients with suspicion of adrenal carcinoma, and patients demonstrating additional adrenal hormone cosecretion.

***AVS procedure/interpretation:*** AVS were performed after correction for hypokalemia and withdrawal of aldosterone receptor antagonists. Sequential sampling of paired plasma aldosterone and cortisol levels from the lower inferior vena cava (IVC), middle IVC, upper IVC, and three samples each from the left then right adrenal veins was performed following a bolus of 250 μg cosyntropin. Results were interpreted according to the expert consensus by Rossi et al. (see Table 1)^15^ An adrenal‐to‐IVC cortisol ratio of >3:1 (selectivity index, SI) defined successful selective adrenal vein cannulation. Bilateral successful cannulation was confirmed, and PA was lateralized if the cortisol‐corrected aldosterone gradient between both sides was >4:1 (lateralization index, LI).

**TABLE 1:** Definitions of adrenal venous sampling indexes in this study and their respective cutoff values.

Definition	Formula	Interpretation
Selectivity index (SI)	${PCC}_{Adrenal}/{PCC}_{IVC}$	Value > 3 confirms that the blood sample was obtained from the adrenal vein
Lateralization index (LI)	$\frac{{PAC}_{Dom}}{{PCC}_{Dom}}/\frac{{PAC}_{Nondom}}{{PCC}_{Nondom}}$	Value > 4 lateralizes aldosterone excess to the dominant side
Contralateral suppression index (CSI)	$\frac{{PAC}_{Nondom}}{{PCC}_{Nondom}}/\frac{{PAC}_{IVC}}{{PCC}_{IVC}}$	Value < 0.5 suggests significant suppression of the normal nondominant side
Relative adrenal secretory index (RASI)	$\frac{{PAC}_{Dom}}{{PCC}_{Dom}}/\frac{{PAC}_{IVC}}{{PCC}_{IVC}}$	Value > 2.4 suggests aldosterone excess is mostly derived from the dominant side

Abbreviations: Dom, dominant side, that is, adrenal gland with largest nodule or highest aldosterone level or positive uptake on scintigraphy; IVC, inferior vena cava; Nondom, nondominant side; PAC, plasma aldosterone concentration; PCC, plasma cortisol concentration.

The adrenal with the largest nodule on CT, highest cortisol‐corrected aldosterone level, or positive uptake on iodocholesterol scintigraphy was denoted as the dominant adrenal. The contralateral suppression index (CSI) was calculated as the ratio of cortisol‐corrected aldosterone levels between nondominant adrenal and IVC. The relative adrenal secretory index (RASI) was calculated as the ratio of cortisol‐corrected aldosterone levels between dominant adrenal and IVC.

Patients were offered unilateral adrenalectomy when AVS demonstrated an LI > 4. When AVS failed at bilateral cannulation, a multidisciplinary discussion took place to resolve the decision for surgery based on clinical, biochemical, computed tomography, and iodocholesterol scintigraphy findings. Following surgery, patients were reviewed at 1, 6, and 12 months postoperation for morbidities, blood pressure control, and biochemistry (serum potassium level, plasma renin activity, and plasma aldosterone concentration).

***Study endpoints:*** The primary endpoint of this study was the diagnostic performance of CSI and RASI from unilateral‐cannulating AVS in predicting uPA. uPA was defined by the biochemical cure of aldosterone excess following adrenalectomy as determined by the normalization of the plasma aldosterone concentration and the ARR at the 12‐month postoperative follow‐up.

***Statistical analysis:*** Results were expressed as mean ± SD or median (IQR). Data were compared using the Mann–Whitney U or Pearson’s X^2^ tests. Statistical significance was set at *p* < 0.05. Sensitivity/specificity plots were performed with jamovi (Version 2.5; the jamovi Project, Sydney, Australia) and the remaining analyses were performed with SPSS (version 29 for Mac; IBM‐SPSS, Bologna, Italy).

## 3 RESULTS

### 3.1 Baseline characteristics

**FIGURE 1:**
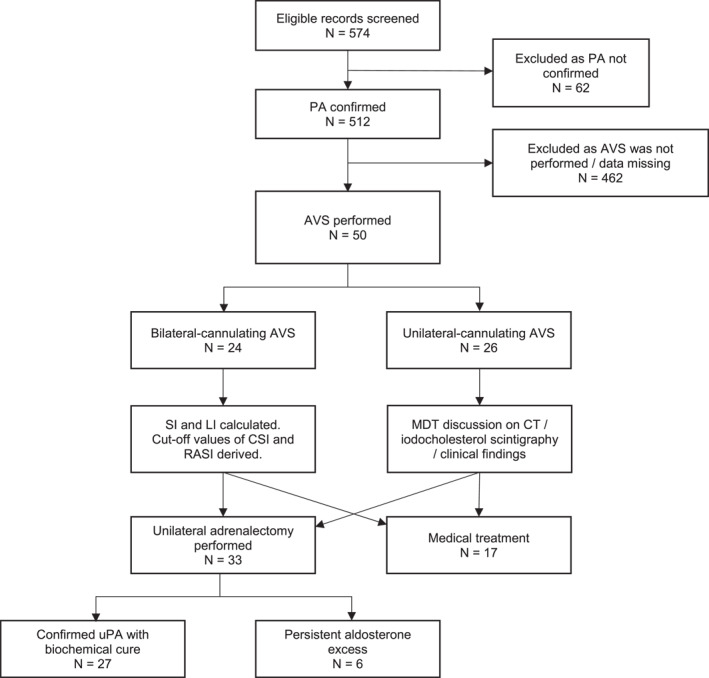
Study flowchart. AVS, adrenal venous sampling; CSI, contralateral suppression index; LI, lateralization index; MDT, multidisciplinary team; PA, primary aldosteronism; SI, selectivity index; and uPA, unilateral primary aldosteronism.

Five hundred twelve patients from 574 screened records were diagnosed with PA. Fifty (9.8%) patients underwent AVS and were included in the study. Figure 1 illustrates the study workflow. Thirty‐six (72%) patients were male, and the median age was 51.5 (IQR: 43–58) years. All patients were hypertensive, 78.0% had concomitant hypokalemia, and one presented with periodic paralysis. The median ARR level was 2268 (IQR1: 333–6943) pmol/L per ng/mL/h. The median 24‐h urine aldosterone level was 74.4 (IQR: 62–101) nmol/d. Table 2 summarizes the study population's baseline demographics and clinical and biochemical characteristics.

**TABLE 2:** Baseline demographic, clinical, and biochemical characteristics.

		Unilateral cannulating AVS		Bilateral cannulating AVS		Total	
		*N* = 26		*N* = 24		*N* = 50	
Sex (male)		16	(61.5%)	20	(83.3%)	36	(72.0%)
Age (years)		51	±9	50	±9	51	±9
BMI >30 kg/M^2^		2	(7.7%)	2	(8.3%)	4	(8.0%)
Present as adrenal incidentaloma		2	(7.7%)	1	(4.2%)	3	(6.0%)
Hypertensive (ambulatory BP > 140/90 mmHg)		24	(92.3%)	24	(100.0%)	48	(96.0%)
Spontaneous hypokalemia		24	(92.3%)	17	(70.8%)	41	(82.0%)
Lowest serum potassium level (mmol/L)		2.70	±0.37	2.922	±0.53	2.81	±0.46
Plasma renin activity (ng/mL/h)		0.40	±0.53	0.24	±0.21	0.32	±0.40
Plasma aldosterone concentration (pmol/L)		705.64	±431.77	590.68	±315.82	648.16	±378.34
Aldosterone–renin ratio at baseline (pmol/L per ng/ml/h)		5168.53	±5982.26	4749.43	±5260.17	4958.98	±5570.95
24 h urine aldosterone level (pmol/d)		75.83	±17.24	73.25	±39.72	74.36	±31.08
24 h urine‐free cortisol (pmol/d)		125.71	±60.30	131.85	±92.14	127.08	±61.61
Computed tomography	No nodules	0	(0.0%)	0	(0.0%)	0	0.0%)
	Bilateral nodules	6	(23.1%)	2	(8.3%)	8	16.0%)
	Unilateral nodules	20	(76.9%)	22	(91.7%)	42	84.0%)
	‐ Right/Left	9/12		9/13		18/25	
Mean size of adrenal nodules (cm)		1.45	±0.54	1.36	±0.59	1.40	±0.56
Nodule density (HU)		9.6	±8.0	16.1	±17.5	12.9	±14.0
I^131^ iodocholesterol scintigraphy uptake	None	3	(20.0%)	1	(25.0%)	4	(21.1%)
	Unilateral	10	(66.7%)	2	(50.0%)	12	(63.2%)
	Bilateral	2	(13.3%)	1	(25.0%)	3	(15.7%)
Paradoxical drop in plasma aldosterone from supine to erect posture		17	(68%)	14	(70%)	31	(68.9%)

Abbreviations: AVS, adrenal venous sampling; BP, blood pressure; BMI, body mass index; HU, Hounsfield unit.

### 3.2 Subtyping of primary aldosteronism

All patients underwent contrast CT. 84% of the CT demonstrated unilateral adrenal nodules and 16% showed bilateral nodules. The average nodule size was 1.45 ± 0.58 cm. Postural stimulation was performed on 45 (90%) patients. A paradoxical drop of plasma aldosterone concentration from the supine to erect posture, which suggested uPA, was observed in 68.9%. Nineteen (38%) patients underwent iodocholesterol scintigraphy, 63.2% of which demonstrated a unilateral hyperfunctioning lesion, 15.8% had bilateral uptake, and 21.1% were negative.

Of the 50 patients with AVS, 44 (88%) had the left, 25 (50%) had the right, and 24 (48%) had bilateral adrenal veins successfully cannulated. No AVS procedure‐related complications were documented.

### 3.3 Adrenalectomy

Following subtyping, unilateral adrenalectomy was performed in 33 (66%) patients, of which 12 were right‐sided and 21 were left‐sided. Fifteen (45.5%) operations were AVS‐lateralized. Of the 18 operations that were not AVS‐lateralized, 15 had unilateral nodule(s) on CT and none were discordant with scintigraphy findings. Three patients had bilateral nodules on CT and underwent unilateral adrenalectomy; one was guided by unilateral uptake on scintigraphy, whereas the remaining two were guided by postural stimulation tests and the adrenal with the dominant nodule was removed. Meanwhile, five patients whose AVS results suggested uPA refused surgery and continued medical treatment.

### 3.4 Post‐adrenalectomy outcomes

Following surgery, hypertension improved in all patients. Seventeen (51.6%) patients demonstrated complete remission of hypertension (ambulatory BP < 140/90 mmHg). The rates of hypertension remission among AVS‐lateralized patients were similar to those who were not AVS‐lateralized (53.3% vs. 50.0% and *p = 0.981*).

At 12 months postoperation, 27 (81.8%) patients had normalized plasma aldosterone concentration and an ARR <750 pmol/L per ng/mL/h representing the biochemical cure. 86.7% of the AVS‐lateralized patients and 77.8% of those not AVS‐lateralized were biochemically cured after adrenalectomy (*p = 0.423*). The median LI among patients who were cured was similar to that of patients with persistent disease (15.71 vs. 46.09 and *p = 0.381*). The median CSI (0.249 vs. 0.282 and *p = 0.889*) and median RASI (4.730 vs. 3.008 and *p = 0.312*) were similar among those cured or with persistent disease. Table 3 summarizes the lateralization results and the postoperative outcomes among patients who underwent adrenalectomy.

**TABLE 3:** Results of lateralization and post‐adrenalectomy outcomes.

	Hypertension remitted			Biochemically cured		
	Yes	No		Yes	No	
Operation:						
AVS‐lateralized (LI > 4)	7	8	*p = 0.981*	13	2	*p = 0.423*
Non AVS‐lateralized	8	8		14	4	
AVS results:						
Median LI	18.8 (14.9–45.0)	15.8 (14.2–43.6)	*p = 0.372*	15.7 (14.3–36.3)	46.1 (15.8–76.4)	*p = 0.381*
Median CSI	0.235 (0.190–0.286)	0.332 (0.174–0.773)	*p = 0.416*	0.249 (0.190–0.449)	0.282	*p = 0.889*
Median RASI	4.53 (3.79–6.27)	4.74 (1.57–10.42)	*p = 0.422*	4.73 (3.18–9.30)	3.01 (1.57–4.45)	*p = 0.312*

*Note*: Italic values indicate the *p*‐values

Abbreviations: AVS, adrenal venous sampling; CSI, contralateral suppression index; CT, computed tomography; LI, lateralization index; MDT, multidisciplinary team; RASI, relative aldosterone secretory index.

Table 4 summarizes the lateralization results and postoperative outcomes of six patients with persistent disease following adrenalectomy.

**TABLE 4:** Patients with persistent hyperaldosteronism following unilateral adrenalectomy.

Age	Postural stimulation tests	CT nodule(s)	Iodocholesterol scintigraphy uptake	AVS bilateral cannulation	RASI	CSI	HT cured	Hypokalemia resolved	ARR <750 pmol/L per ng/ml/h	PAC normalized	PRA normalized
62	Bilateral hyperplasia	Bilateral (left 2.9 cm and Right 6 mm)	Left	Yes, LI = 76.4	7.53	0.0985	No	Yes	No	Yes	No*
56	Unilateral PA	Bilateral micronodules	Negative	No	0.28	‐	No	No	No	No	No
54	Unilateral PA	Left 1.7 cm nodule	Left	No	5.84	‐	No	Pre‐op normal	No	Yes	No*
53	Unilateral PA	Left 1.9 cm nodule	Negative	No	1.57	‐	Yes	Pre‐op normal	No	No	No
53	Unilateral PA	Right 1.9 cm nodule	Negative	Yes, LI = 15.8	4.45	0.2822	No	Pre‐op normal	No	Yes	No*
63	Bilateral hyperplasia	Right 1.7 cm nodule	Right	No	Failed cannulation	Failed cannulation	No	Yes	No	No	No

Abbreviations: ARR, Aldosterone‐renin ratio; AVS, adrenal venous sampling; CT, computed tomography; CSI, contralateral suppression index; HT, hypertension; LI, lateralization index; PA, primary aldosteronism; PAC, plasma aldosterone concentration; PRA, plasma renin activity; RASI, relative aldosterone secretory index.* Plasma renin activity suppressed potentially by use of antihypertensive agents, for example, angiotensin converting enzyme inhibitors, aldosterone receptor modulators, direct renin inhibitors, and beta‐blockers.

### 3.5 CSI and RASI cutoffs

We derived the optimal threshold of CSI and RASI from 24 patients with bilateral cannulating AVS. The median CSI was 0.25 (IQR: 0.12–0.46) in patients with LI > 4 versus 2.68 (IQR: 0.55–2.90) in patients with LI < 4 (*p* = 0.01). The median RASI was 4.64 (IQR: 3.79–10.21) in patients with LI > 4 versus 2.13 (IQR: 0.28–2.42) in patients with LI < 4 (*p* = 0.01).

**FIGURE 2:**
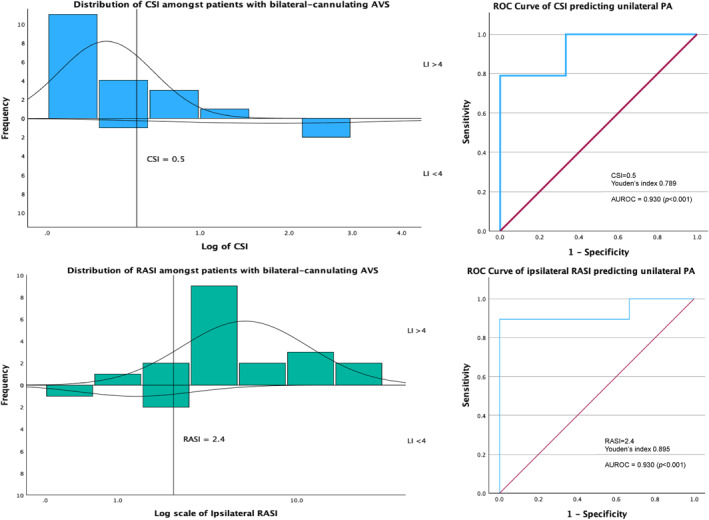
Distribution of CSI and RASI in patients with bilateral cannulating AVS. ROC analysis for optimal cutoff values.

Receiver‐operating characteristic (ROC) analysis of CSI and RASI demonstrated the optimal cutoff values as CSI <0.5 (Youden's index 0.789) and RASI >2.4 (Youden's index 0.895), respectively. Figure 2 illustrates the distribution of CSI and RASI among patients with bilateral cannulating AVS.

### 3.6 Predicting biochemical cure of PA following adrenalectomy

Table 5 summarizes the diagnostic performance of the postural stimulation test, CT, iodocholesterol scintigraphy, and AVS in identifying uPA.

**TABLE 5:** Diagnostic performance of different methods to subtype PA in predicting uPA as defined by biochemical cure following adrenalectomy.

Subtyping method	Sensitivity (95% confidence interval)	Positive predictive value (95% confidence interval)
Bilateral AVS with LI > 4	100.0 (75.3–100.0)	86.7 (59.5–98.3)
Computed tomography	92.6 (75.7–99.1)	86.2 (68.3–96.1)
Postural stimulation test	70.8 (48.9–87.4)	81.0 (58.1–94.6)
I131 iodocholesterol scan	50.0 (18.7–81.3)	62.5 (24.5–91.5)
Unilateral AVS with CSI <0.5	76.5 (50.1–93.2)	92.9 (66.1–99.8)
Unilateral AVS with RASI >2.4	85.0 (62.1–96.8)	94.4 (72.7–99.9)
Unilateral AVS with either CSI <0.5 or RASI >2.4	87.5 (67.6–97.3)	95.5 (77.2–99.9)

Abbreviations: AVS, adrenal venous sampling; CSI, contralateral suppression index; LI, lateralization index; PA, primary aldosteronism; RASI, relative aldosterone secretory index; uPA, unilateral primary aldosteronism.

Bilateral AVS with an LI > 4 provided a sensitivity of 100% (95% CI 75.3–100.0) and a positive predictive value (PPV) of 86.7% (95% CI 59.9–98.3) for uPA. CSI of <0.5 would have provided a sensitivity of 76.5% and a PPV of 92.9%, whereas RASI of >2.4 would have given a sensitivity of 85.0% and a PPV of 94.4%.

With either CSI <0.5 or RASI >2.4 combined as one criterion, the diagnostic performance for uPA would have been significantly better than iodocholesterol scintigraphy at identifying uPA (sensitivity of 87.5% vs. 50.0%, *p* = 0.021 and PPV of 95.5% vs. 62.5%, *p* = 0.019), and also noninferior to CT (sensitivity of 87.5% vs. 92.6%, *p* = 0.545 and PPV of 95.5% vs. 86.2%, *p* = 0.276).

## 4 DISCUSSION

The AVS results directly determine the patients who are selected for surgery and their outcomes. The 2008 Endocrine Society guideline recommended an SI > 10 with cosyntropin for defining cannulation success and an LI > 4 for determining lateralization. ^16^ However, an international survey reported that 60% of centers used an SI cutoff of 3–5 with cosyntropin stimulation. ^10^ Generally, a lower SI cutoff will reduce the diagnostic accuracy of AVS. In contrast, a higher cutoff will exclude a portion of otherwise successful AVS from diagnostic use. These are also true for the LI cutoff used to determine lateralization. Few centers have prospectively evaluated the optimal cutoffs for AVS interpretation. ^17^ As no data are available concerning the optimal cutoffs in our population, we followed the international expert consensus by Rossi et al., where an SI > 3 with cosyntropin confirms cannulation success and an LI > 4 confirms lateralization. ^15^ These cutoffs must be considered when results from this study are interpreted and compared with others.

Our study justified adopting these cutoffs by demonstrating a high PPV (86.7%) of bilateral AVS in identifying uPA, equating to a high biochemical cure rate following AVS‐guided adrenalectomy. Most studies on post‐adrenalectomy outcomes have ill‐defined criteria and variable follow‐up. ^8^, ^18^, ^19^, ^20^, ^21^, ^22^, ^23^ We adopted the consensus definitions of postoperative outcomes from the Primary Aldosteronism Surgical Outcome (PASO) study. Our results are comparable to the international cohort's 94% (95% CI 83%–100%) biochemical remission rate. ^24^

AVS success is associated with expertise and volume. Our study achieved bilateral cannulation in only 48% of patients, which is comparably lower than the 78%–90% success rate reported by other groups in our locality. ^25^, ^26^ Nonetheless, up to 88% of patients have at least the left adrenal vein successfully cannulated. Moreover, the absence of AVS‐related complications shows that AVS is safe and feasible even in low‐volume centers.

When bilateral cannulation cannot be achieved, various reports have subtyped PA by assessing the contribution of a single adrenal to the overall plasma aldosterone level. Wolley et al. and Umakoshi et al. defined suppressed contralateral adrenal secretion as a CSI <1, which correlated with biochemical cure and resolution of hypertension following adrenalectomy. ^11^, ^18^ However, others described various CSI cutoffs ranging from 0.5 to 1.4. ^23^, ^27^, ^28^ Pasternak et al. reported an RASI ≥5.5 which provided a PPV of 100% for uPA. ^23^ However, Strajina et al. contended that RASI ≥5.5 would result in inappropriate adrenalectomy in 18% of patients and miss 55% of patients with uPA. ^29^ Currently, consensus on cutoffs for CSI or RASI is absent. In this study, CSI <0.5 and RASI >2.4 were the best at stratifying patients with bilateral cannulation into those with LI > 4 and < 4. These cutoffs were then applied to the entire study cohort. The advantage of these cutoffs is that the decision based on the CSI or RASI would least likely contradict the decision based on the LI from bilateral AVS in the same individual.

In this study, lateralizing PA by CSI <0.5 or RASI >2.4 combined provided a high sensitivity (87.5%) and PPV (95.5%). Although Strajina et al.^29^ reported poor diagnostic performance with an RASI ≥5.5, others, such as Rossi et al.,^28^ reported favorably with an RASI >2.5. When interpreting these studies, we must acknowledge that although some authors defined uPA by the conclusion of bilateral AVS, our study defined uPA by the biochemical remission following surgery. This may explain why a lower RASI cutoff can be utilized.

Although the PPV of unilateral AVS was significantly better than that of iodocholesterol scintigraphy, no statistically significant difference was demonstrated between the PPV of unilateral AVS, bilateral AVS, and CT. Firstly, this study may be underpowered due to the limited sample size. Furthermore, as screening for PA in hypertensive patients is not widely practiced in Hong Kong, a higher proportion of patients were detected with more severe disease and with more incidental adrenal nodules on imaging. This may skew the prevalence of aldosterone‐producing adenoma in our cohort and therefore, inflate the PPV of CT.

Based on our results, we did not demonstrate that bilateral AVS was superior to unilateral‐cannulating AVS for identifying uPA. However, as aforementioned, a diagnostic conclusion based on bilateral AVS would unlikely contradict unilateral‐cannulating AVS due to the method by which cutoffs were derived in our study. Yet, when bilateral cannulation cannot be achieved during AVS, our study demonstrates that unilateral‐cannulating AVS was accurate in selecting patients for surgery. Only when unilateral‐cannulating AVS disagreed with CT would it have been prudent to consider repeat AVS.

Finally, our study had several limitations. The sample size was small, and the study period was protracted. A larger multicenter design might yield greater statistical power and better compare the diagnostic performance of unilateral AVS and CT. However, despite the long study period, the AVS procedures were consistently performed using a standardized technique and interpretation protocol. The low case volume is also considered a strength of this study because it better reflects real‐life scenarios and is more generalizable to small‐volume centers that most benefit from using unilateral‐cannulating AVS results to guide adrenalectomy.

## 5 CONCLUSION

AVS at low‐volume centers often fail at bilateral adrenal vein cannulation. Yet, unilateral‐cannulating AVS is safe and can effectively select patients for unilateral adrenalectomy. AVS should be encouraged outside of expert centers.

## AUTHOR CONTRIBUTIONS

**Chi‐Man Tom Chow**: Conceptualization; Data curation; Formal analysis; Methodology; Writing ‐ original draft; Writing ‐ review and editing. **Man Sze Carol Lai**: Validation. **Xina Lo**: Supervision. **Yuk Wah Shirley LIU**: Resources; Supervision; Writing ‐ review and editing.

## CONFLICT OF INTEREST STATEMENT

The authors declare that they have no conflicts of interest.

## ETHICS STATEMENT

The study has obtained ethics approval from the Chinese University of Hong Kong Clinical Research Ethics Committee/Institutional Review Board.

## CONSENT

Informed consent was obtained from all participants included in the study.

## Data Availability

The data that support the findings of this study are available from the corresponding author upon request.
